# Malpractice claims for pancreatic cancer in Norway: claim-rates, injury domains, claim outcomes and indemnity compensation

**DOI:** 10.2340/ao.v65.45500

**Published:** 2026-04-13

**Authors:** Annbjørg H. Søreide, Karin H. Edland, Andreas B. Alvestad, Solveig Hodne, Kjetil Søreide

**Affiliations:** a Lervig Legesenter, Stavanger Municipality Primary Healthcare, Stavanger, Norway; b Department of Oncology, Stavanger University Hospital, Stavanger, Norway; c Department of Radiology, Stavanger University Hospital, Stavanger, Norway; d Stavanger Medical Imaging Laboratory (SMIL), Stavanger University Hospital, Stavanger, Norway; e SHARE-Centre for Resilience in Healthcare, Faculty of Health Sciences, University of Stavanger, Stavanger, Norway; f Department of Quality and Health Technology, University of Stavanger, Stavanger, Norway; g Department of Gastrointestinal Surgery, Stavanger University Hospital, Stavanger, Norway; h Department of Clinical Medicine, University of Bergen, Bergen, Norway; i Department of Surgery, Institute of Clinical Sciences, Sahlgrenska Academy, University of Gothenburg, Gothenburg, Sweden

**Keywords:** Pancreatic cancer, malpractice, litigation, management errors, compensation

## Abstract

**Background and purpose:**

Pancreatic cancer is difficult to diagnose early and at a curative stage, yet little is known about errors in management. The aim of the study was to investigate malpractice claims in Norway.

**Patients and Methods:**

All malpractice claims filed to the Norwegian System of Patient Injury Compensation between 2015 and 2024 for pancreatic cancer were evaluated.

**Results:**

A total of 148 claims (median 15 [range 8–20] claims/year) were filed among 9548 patients with pancreatic cancer, for an average claim rate of 1.55% (1 claim per every 65 pancreatic cancers). Most claims were filed against hospital/specialists (*n* = 90), followed by general practitioners (*n* = 49) with nine claims towards private practice contractors (*P* < 0.001). A total of 33 claims (22.3%) were approved, of which 28 (85%) concerned delayed diagnosis. Radiology was involved in 18 of the 33 approvals. According to caretaker-level for the malpractice claim, approved claims were 23% for hospital-claims, with lowest approval-rate against general practitioners (12%), and highest against private contractors (66%). Median delay in diagnosis was estimated at 6 months. The outcome of the error was ‘death’ in 16 of 33. Prognostic loss was determined in five of 33 patients, while 12 patients had no prognostic loss despite an approved claim. Caretaker-level did not differ regarding death or prognostic loss in claims (*P* = 0.203). A total of 1.47 million EUR was paid in indemnity payments.

**Interpretation:**

The claim rate of 1.5% was stable, with about one in five claims approved. Diagnostic delay was the predominant cause, with several stakeholders involved across the healthcare system.

## Introduction

Pancreatic cancer is difficult to diagnose at an early and operable stage [1–3]. Consequently, pancreatic cancer has a high lethality rate and one of the worst long-term survival rates among solid organ tumours and, is estimated to become the second leading cause to cancer deaths globally within the next few years. Among all patients presenting with pancreatic cancer, a mere 15–20% can be offered curative attempt-surgery with optimal multimodal systemic therapy [4, 5]; as many as 40–50% of all patients may not receive any form of active treatment, either due to higher age and frailty or due to locally advanced or disseminated disease [4, 5].

Pancreatic cancer presents with few or rather unspecific and vague symptoms in its early stage (i.e. unspecific abdominal discomfort; non-specific symptoms), with more prominent or overt clinical signs (i.e. weight-loss and cachexia; jaundice; abdominal or back pain) usually indicating more advanced and often incurable disease [1]. Hence, the clinical diagnosis represents a considerable challenge for clinicians. Referral of patients into urgent care pathways for diagnosis have been implemented and proposed across healthcare systems, however, with variation in use and interaction between primary care and hospital specialties [6, 7]. The risk of having advanced disease at time of diagnosis or, being unfit for any active treatment (surgery or chemotherapy) increases with age [8]. The concept of error in cancer diagnostics is not new [9], but evaluation and analyses of malpractice claims has been done only for a few malignancy types in the past [10, 11] or only in relation to one medical specialty, such as medical oncologists [12], rather than the entire care pathway as such. Also, many healthcare systems have a litigation and court system that is based on blame and is primarily kept in the court system [12, 13].

In Norway, the healthcare response to malpractice claims is based on a ‘no blame system’, and indemnity payments rather based on the degree of injury or prognostic loss. The objective of this study was to describe and analyse all claims filed for pancreatic cancer across an entire nationwide cohort over a decade, to understand the frequency, types and outcomes of such malpractice claims made for pancreatic cancer.

## Materials and methods

### Study’s ethics

The study was based on administrative data delivered in anonymized form and as provided from the administrative office of the Norwegian System of Patient Injury Compensation (Norsk Pasientskadeerstatning, NPE). The NPE data were extracted from the internal files only available to NPE employees. All identification and anonymization of files were performed by the NPE staff. The anonymized and aggregated files were made available after signed confidentiality contract with NPE.

No formal ethical application and approval was deemed required for the study, based on project evaluation put forward to the Regional Ethics Committee of the Western region in Norway (REK-Vest #915996).

### Study cohort of pancreatic cancer and cancer pathways

The data-sources for the annual frequency of pancreatic cancer were based on registered annual new events as presented in the most updated report Cancer in Norway for 2024, with no ethical approval required for usage, as data are anonymized, nonidentifiable and presented as aggregated numbers.

### Study setting

The study was done in a single-payer, universal healthcare system in Norway. This nationwide study investigates malpractice claims across the four health authority regions (i.e. the specialist hospital care provided by hospitals) as well as claims to municipal care and primary healthcare setting (i.e. emergency clinics and general practice).

The Norwegian health care system holds a strong position and tradition on the primary healthcare, with considerable roles for the general practitioner as a gatekeeper to the specialist healthcare system.

Limited private practice exists and in principle concerns private corporate centres offering diagnostic procedures (i.e. radiological imaging) for which the regional health trust may have contracted slots (i.e. for imaging studies) in diagnostic pathways or for follow up.

### The Norwegian system of patient injury compensation

The Norwegian Compensation is in existence since 1988 and follows the principles of ‘no blame’ [14]. The criteria that must be in place for a claim to be evaluated in general follows the current judicial assumption of patient injury according to the Law of Patient Injury (Pasientskadeloven) as applied to all public healthcare in 2003 and all private healthcare in 2009. In brief, this includes the following requirements.

1. **The patient injury must be due to treatment failure:** The complainant must have suffered an injury that was a direct consequence of the treatment, examination, diagnosis or follow-up (i.e. causality between claim and consequence). The patient injury may be temporary or permanent. In addition, the injury must be due to an error or omission in the treatment. If the injury is a result of the very illness the patient or complainant was treated for, there will be no entitlement to compensation. An injury sustained, which was not caused by an error or omission in the treatment, will not normally be entitled to compensation to the complainant. In some exceptional cases (‘rule of exemption’), the complainant may be awarded compensation even if there has not been an error or omission in the treatment. This applies to injuries that are particularly severe and unexpected.
2. **The patient injury must have resulted in financial loss:** As a rule, the patient injury must have resulted in financial loss in addition to any losses that would otherwise have been incurred. The purpose of the compensation is to cover additional expenses incurred because of the patient injury. This could include expenses for medical treatment, medication, transport or compensation for loss of income or loss of provider. However, for a financial loss < 10.000 NOK (or, approximately < 1,000 EUR), compensation from NPE cannot be claimed.
3. **The patient injury must not be too old:** A complainant can apply for compensation for a patient injury for up to 3 years after they ought to have understood that it was the treatment received or a lack of treatment, which caused the injury. If you apply after 3 years, the claim will, as a general rule, be considered to be expired.

### Study period and inclusion

All claims filed to NPE for the diagnosis of ‘pancreatic cancer’ (ICD-10 codes C25.x) between January 2015 and December 2024 were requested for review. The fiscal decade represents the NPE year by which a verdict of the claim was filed. As a claim can be filed up to 3 years after an incident, and thus there is no immediate overlap with the annual incidence per se. Due to the immediacy and overall short-term survival in pancreatic cancer, a relative concordance in annual claims to cancer incidence is assumed. The period was split into early and late to explore any difference over time-periods (2015–2020 and 2021–2024).

### Statistical analysis

Statistical analyses were done using SPSS for Mac v 29. Categorical variables were analysed by 2 × 2 tests using chi-square or Fisher’s exact test. All statistical tests were two-tailed and significance attributed to *P* < 0.050.

## Results

**Figure 1:**
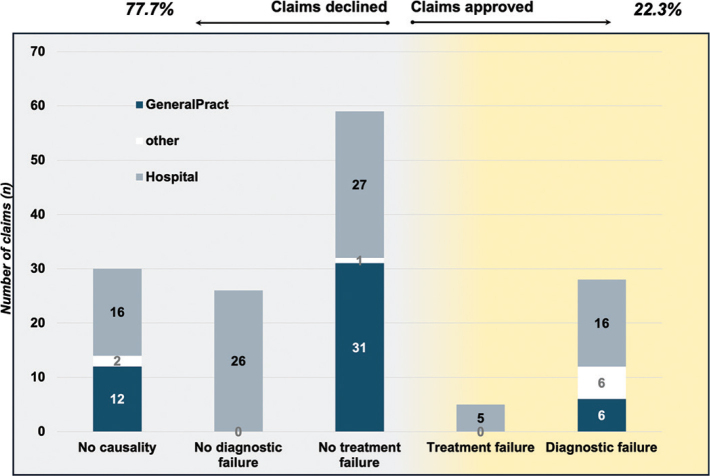
New cases of pancreatic cancer events (A) and claim-rates over time (B). The source for annual cases in (A) is delivered from the Cancer Registry of Norway, while the claim rate (B) is based on annual claims in NPE.

Over the 10-year study period there were 148 claims, with a median of 15 (range 8–20) claims/year. A total of 9,548 new episodes of pancreatic cancer occurred between 2015 and 2024, for an average claim rate of 1.55% of all pancreatic cancer diagnosis or, with an episode-claim-rate of one claim per every 65 events of pancreatic cancer (Figure 1A, B). There were no differences in claim approval between the two time-periods, nor in distribution of claims in health regions, nor in age-groups, but an increase in male rate from the first period (male rate first period 48% vs. male rate second period 64%; *P* = 0.049).

### Characteristics of claims filed to NPE

**Figure 2:**
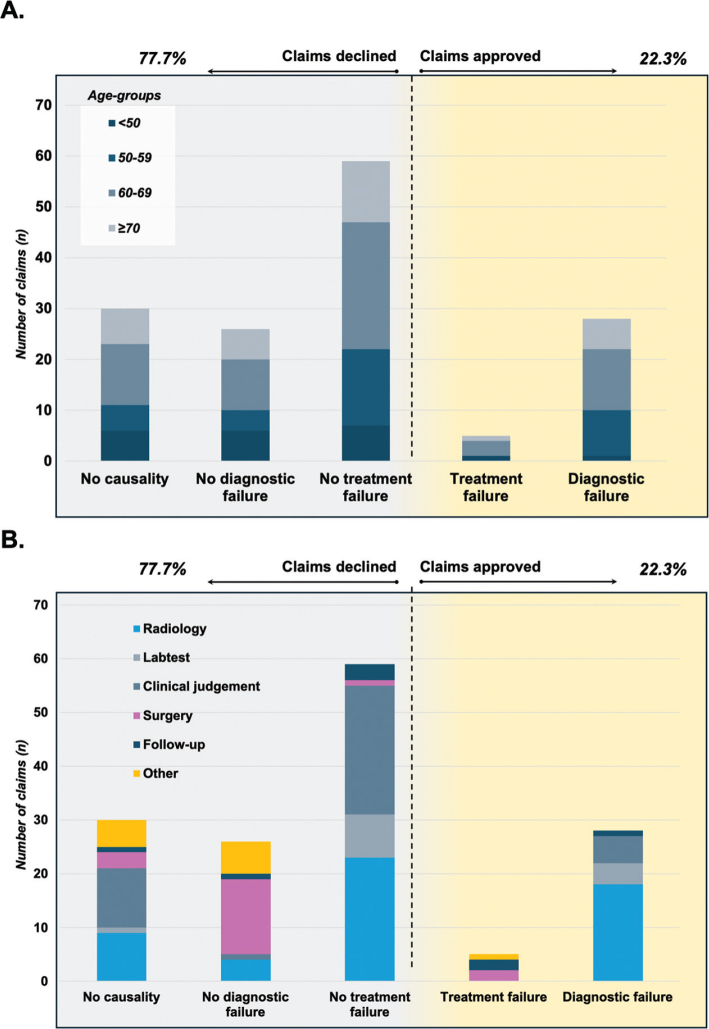
Characteristics for claims filed across age-groups and for source of malpractice claim. (A) according to age-groups. (B) according to claimed source of the malpractice.

The characteristics of the claims are presented in Table 1. No differences were found for distributions in gender for outcome of claims, nor for the assigned management category of the injury for the claims. Distribution between age-groups and the perceived source of the management error for the claims are presented in Figure 2A, B.

**Table 1:** Characteristics among the malpractice claims filed in Norway over a decade

	Total	Declined claims	Approved claims	*P*
**N**	148	115	33	
**Sex, *n* (%)** Male Female	84 (56.8%) 64 (43.2%)	65 (56.5%) 50 (43.5%)	19 (57.6%) 14 (42.4%)	*0.914*
**Age-groups, *n* (%)** < 50 years 50–59 years 60–69 years ≥ 70 years	21 (14.2%) 33 (22.3%) 62 (41.9%) 32 (21.6%)	19 (16.5%) 24 (20.9%) 47 (40.9%) 25 (21.7%)	2 (6.1%) 9 (27.3%) 15 (45.5%) 7 (21.2%)	*0.461*
**Cancer location/type, *n* (%)** Head Body Tail Unspecified location Neuroendocrine	52 (35.1%) 10 (6.8%) 12 (8.1%) 68 (45.9%) 6 (4.1%)	36 (31.3%) 7 (6.1%) 10 (8.7%) 57 (49.6%) 5 (4.3%)	16 (48.5%) 3 (9.1%) 2 (6.1%) 11 (33.3%) 1 (3.0%)	*0.367*
**Healthcare level for claim** Specialist healthcare Primary healthcare Other*	90 (60.8%) 49 (33.1%) 9 (6.1%)	69 (60.0%) 43 (37.4%) 3 (2.6%)	21 (63.6%) 6 (18.2%) 6 (18.2%)	*< 0.001*
**Management category for claims** Radiology/imaging Laboratory test Clinical judgment Surgery Follow up Other	54 13 41 20 8 12	36 (66.7%) 9 (69.2%) 36 (87.8%) 18 (90%) 5 (62.5%) 11 (91.7%)	18 (33.3%) 4 (30.8%) 5 (12.2%) 2 (10%) 3 (37.5%) 1 (8.3%)	*0.053*
**Time period of claim** 2015–2020 2021–2024	65 83	52 (80%) 63 (75.9%)	13 (20%) 20 (24.1%)	*0.552*

* Other, includes healthcare services bought from public healthcare (*n* = 7) and specialists in private practice (*n* = 2).

### Claims approved

Some 33 claims were approved, of which 28 (85%) concerned delayed diagnosis for various reasons. Three of the approved claims were for erroneous management (i.e. bowel obstruction; bleeding related to surgical procedures in 2); one for wrong medication and one for untimely/delayed medication (for a cerebral stroke).

The outcome of the error was ‘death’ in 16 of 33; most were related to hospital management (10 of 21), with five (of six) related to general practitioners’ care and one of 3 related to private contractors. Prognostic loss was determined in five of 33 patients, and 12 patients were determined to have had no prognostic loss despite an approved claim. There was no statistical significance in the rates between the caretaker level (*P* = 0.203) regarding death or prognostic loss.

**Figure 3:**
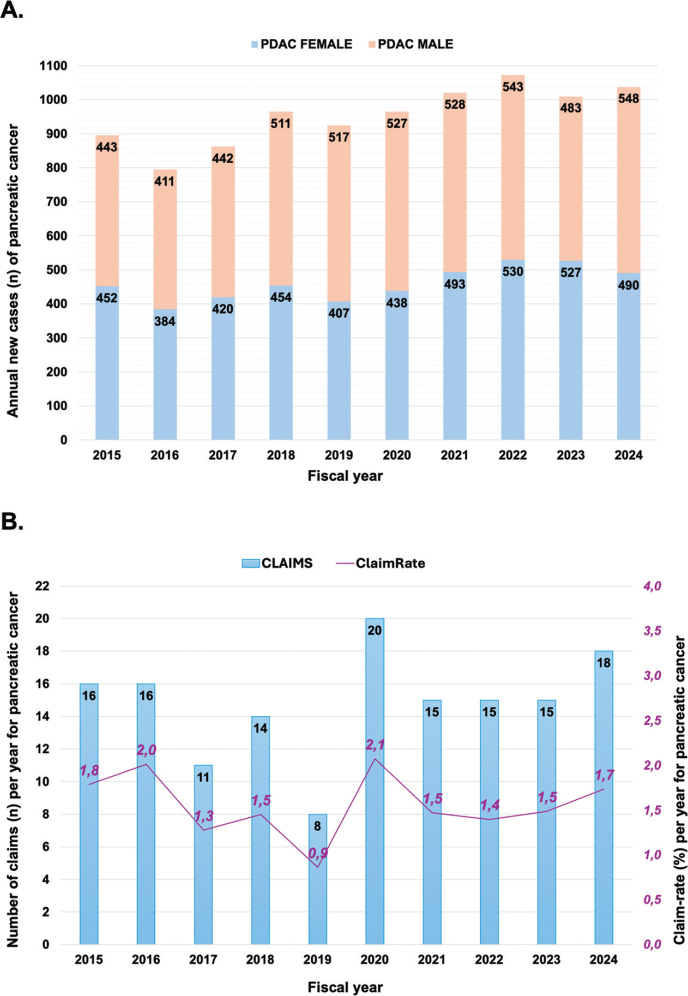
Outcome of claims based on the level of care giver. Numbers in columns are cases per care provider level within the groups of declined/approved claims.

Radiology or imaging was involved in 18 of the 33 approved claims, and all concerned delay in diagnostics in some form. The caretaker level was general practitioners in two of 18 (Figure 3), private contractor in six and hospital-based care in 10 of 18 events. When time in delay was reported, the median delay in diagnosis was estimated at 6 months, with 2–3 months as the shortest estimated diagnostic delay and as much as 11 years delay in one case. Only four patients had a delay estimated longer than 1.5 years. An error in interpretation of imaging was deemed to be the cause in nine events, most often found with private contractors (five of nine) and in hospital (*n* = 3) and one in general practice (failure to refer finding to specialist care). Six delays concerned findings that warranted further diagnostic workup or other imaging modality; one concerned use of wrong modality of investigation; one concerned failure in communication (at hospital level) and one a delay in referral from the general practitioner. The cases with the longest diagnostic delay (4, 8 and 11 years) all concerned premalignant conditions that should have had earlier diagnostic follow up and in one case MR scan rather than CT.

The four claims approved concerning laboratory tests had an estimated a 5- and 7-months delay in diagnosis in two patients, and 7- and 11-years long delays in further two, respectfully. All of them concerned follow up of symptoms including abdominal pain, diabetes (*n* = 1) and in one case abnormal liver function tests with a 11-year past medical history.

Error in clinical judgement resulted in approved claims in five instances (Table 1). Three of these concerned general practitioners not responding to patients’ symptoms with appropriate imaging studies, and two were hospital-based errors in judgements of which one a declined referral in the cancer pathway and one a delay in diagnostic work up.

Surgery-related claims were approved in two events, one due to a post-operative bleeding and death that was interpreted to be due to the technical closure of the pancreatic stump; a second case was approved based on failed biliary drain management, which resulted in severe post-operative complications and premature death.

Three patients had approved claims due to errors in follow up, all were related to hospital care. The first patient was concerned about a delay in diagnosis of recurrence based on symptoms and suboptimal chemotherapy given; the second patient was complained about an extended stop in anticoagulation prior to a biopsy leading to a cerebral stroke, and the third one was concerned about failed post-operative surveillance leading to complications and a prolonged hospital stay.

Lastly, one claim was approved due to failure to stop anticoagulation prior to biliary stenting with subsequent bleeding and need for splenectomy.

### Indemnity and expenses paid

As of December 5^th^, 2025 for indemnity closures and cases solved, a total indemnity compensation of 16.8 million NOK (average sum of 525,000 NOK; median sum of 187,000 NOK) was paid to approved claims. This translates to an equivalent of (exchange rate as of January 29^th^, 2025) total 1.47 million EUR in indemnity payments. The average expense paid per case of 44,990 EUR per case, for a median 16,381 EUR per case.

## Discussion

This study found a relatively stable claim-rate among patients with pancreatic cancer over the study period. Among all those who filed a claim, some 22% of claims were approved. Most claims were filed for in the diagnosis categories and delays were presented as the main cause of the claims file. This was similar in both approved and denied claims. Radiological diagnostics, with subsequent delays due to either failure to diagnose the disease or due to delays in communication were recurring reasons for claims approvals.

To the best of our knowledge, this is the first malpractice and indemnity study done specifically for pancreatic cancer, in particular for the Nordic countries, and we have not found any similar studies in the available international literature, to our surprise. At the onset of this study, we noted the lack of malpractice and indemnity reports for pancreatic cancer, as this disease is known for being difficult to diagnose and to detect at an early stage, with only a minority eligible for curative attempt treatment. It may indeed be, that many patients are too sick, too old or too frail to consider any claims at the time when they receive the diagnosis. However, it may also be that an inherent trust in healthcare deflects any thoughts of filing a claim. As seen in the current cohort, some 22% of claims were approved, suggesting that most claims did not meet the criteria. To maintain trust, transparency and preferable a low threshold for claims, it is wanted to have more claims than approvals, so that patients may have a sense of a secondary evaluation of the management received. It may very well be though, that there are a number of patients who should have filed a claim, but never did so, and this is neither evaluated in the current cohort nor in the NPE system as such. How well the patients are aware of their own rights and are aware of the criteria for filing claims in the healthcare system is poorly understood and not well investigated. Hence, there may be an underreporting of malpractice in that regard. However, the notion that a high rate of claims in the current setting concerns diagnostic errors and diagnostic delay and involving radiology, is corresponding with other literature on the role and severity of misinterpretation in radiology, and particularly for oncological diagnosis [9, 15].

An obvious difference in claims directed at level of care warrants some comments. Most claims were filed against hospital-caretakers, with one in every five approved; claims directed at general practitioners had a lower approval rate (at 12% of all claims), while private contractors (usually private-institutions providing radiology services) had a very high approval rate at 66%. The latter may be due to the expectations of a very specific task (diagnosis and reporting back to the referring level) done in a high-volume, high-turnover setting expected to deliver specific tasks. Of note, reimbursements pattern and travel times may influence the role and degree of private-service delivery [16], as there is an ongoing pressure on the healthcare systems with timely delivery of diagnostic services in the urgent care pathways, with radiology as a bottleneck at many institutions. The lower rate of approval for claims towards general practitioners may not be surprising, given the overall unspecific symptoms and limited range of diagnostic tests that are available for pancreatic cancer. Hence, the general practitioners are not to blame for the natural course of the disease, even if some patients may feel that any misdiagnosis or malpractice is directed towards the primary caretaker. Nonetheless, clinical vigilance is required to avoid delays or misinterpretations in communication between primary care and specialists.

All five claims approved for treatment malpractice concerned in-hospital management, but notably the type of error or approvals of claims were not all immediately directed to care of pancreatic cancer as such. Some claims included surveillance and other management, including anticoagulation and two cases of complications from surgery. It is noteworthy that post-surgical surveillance has not been recommended as a standard in Norway, despite reported differences in this practice between Nordic countries [17]. In addition, neither single malpractice nor any approved claim was attributed to medical oncology care (i.e. too much or too little treatment; or, for toxicity related to oncological treatment), which may reflect the cause-and-effect perception as well as expectations among patients. Some of the interpretation of cases rests on a subjective evaluation by one expert (i.e. for the choice of surgical closure of the pancreatic stump, for which there are many technical solutions with no clear superiority, but equally many strong opinions by experts). There are no data on any claims that were not approved by NPE that went on to have an appeal in the appeal system (Pasientklagenemda) or, even went to any civil lawsuit, which may occur in some instances.

Another aspect to consider is the claim rate as such, and potential differences between different disease-groups across the healthcare system. It may simply be that some diseases have a lower claim rates per disease/procedure event than others, as seen also for acute pancreatitis [18] and for acute appendicitis [19], both of which have a claim-rate around 0.16% (or, one claim per every 620 appendectomy; or one claim per every 720 event of acute pancreatitis) – around 10 times lower than for pancreatic cancer. In contrast, the claim-rate for hip arthroplasty in Norway is somewhat higher than for pancreatic cancer, reported at 1.9% of all hip arthroplasty procedures [20]. The claim rate for liver conditions in the United States has been reported to be much lower (at 0.09%) [21]. Direct comparison to other cancers or premalignant conditions may prove difficult, for example, for cervical cancer, this would only concern women, of a different age-group (most < 40 years of age), and a majority of claims concerning screening and early detection [10]. It is worth mentioning that for this particular group of younger, female patients with precancer or cancer, the approval rate was 3-times higher than for pancreatic cancer, reported at approvals of 62% of the claims [10].

Some limitations to the study may be mentioned. Firstly, we had no access to the full files of the individual cases and hence it was not possible to explore further details into individual case-based reasons for the various claims, causes and the outcomes. The cases are hence judged on the claims filed, their respective codes in the NPE system and the summary information provided for the case, the claims and the outcome as viewed by the NPE specialist experts. Furthermore, we have no insight into any potential malpractice patterns out with the NPE files, that is, any diagnostic delays for which no claim was filed, despite erroneous practice. Hence, the study only allows investigation into any claims that were actually filed during the study period, without any assumption that practice was otherwise perfect or acceptable for any non-filed incidents. The psychology and societal expectations behind who will file a claim and who will not file is a complex issue that is neither well studied nor very well understood. In general, one can assume that Norway has, at least in the past, had the benefit of a rather great trust in the public healthcare and limited activity in lawsuits and claims of inappropriate care compared to other healthcare systems. Whether this attitude will remain stable or change with public expectations to what healthcare should deliver remains to be investigated.

Disparities in management and outcomes in patients with pancreatic cancer may stem from several sources, largely depending on type of healthcare system [22]. In Norway, as the rest of the Nordic countries, access to healthcare is universal and based on premises of equal access to care. Still, there may be inherent disease attributes that make management prone to suboptimal care if not blatant errors. The difficulty in diagnosing pancreatic cancer overall [23], especially on general cross-sectional scans outside specialized centers is well-known, let alone providing the correct staging when such cancer is present [24]. Modern technology such as artificial intelligence (AI)-enhanced pattern recognition in CT-scans may help avoid making mistakes in the diagnostic process of pancreatic cancer [25]. Indeed, this has now been demonstrated by the non-inferiority PANORAMA study [26], showing equal outcomes by AI-detected cancers to expert radiologists. Such diagnostic enhancement tools may be implemented in the pathway by private-contractors providing out-of-hospital radiology services in order to reduce the failure of certain diagnoses, but even so in non-specialized hospitals, as not all hospitals have specialized, focused pancreatic-disease oriented gastrointestinal radiology services.

## Conclusion

In this nationwide study, the claim-rate for pancreatic cancer was rather stable around 1.55% of cases, with most cases attributed to diagnostic issues and delays, involving several stakeholders across the healthcare system. Importantly, diagnostic delays and failures occur at several levels and remains an Achilles heel in the management of pancreatic cancer.

## Data Availability

Data are available only on application to the NPE, as cases are subject to personal information and requires a non-disclosure statement to be signed.
